# Association of Gut Microbiota Composition with Stunting Incidence in Children under Five in Jakarta Slums

**DOI:** 10.3390/nu16203444

**Published:** 2024-10-11

**Authors:** Ratnayani, Badriul Hegar, Diana Sunardi, Fadilah Fadilah, Hartono Gunardi, Umi Fahmida, Dhanasari Vidiawati

**Affiliations:** 1 Department of Nutrition, Faculty of Medicine, Universitas Indonesia—Dr. Cipto Mangunkusumo General Hospital, Jakarta 10430, Indonesia; ratnayani1105@binawan.ac.id (R.); diana_sunardi@yahoo.com (D.S.); umifahmida@gmail.com (U.F.); 2 Nutrition Study Program, Faculty of Health Sciences and Technology, Binawan University, Jakarta 13630, Indonesia; 3 Department of Child Health, Faculty of Medicine, Universitas Indonesia—Dr. Cipto Mangunkusumo Hospital, Jakarta 10430, Indonesia; h_gunardi@yahoo.com; 4 Department of Chemistry, Faculty of Medicine, Universitas Indonesia, Jakarta 10430, Indonesia; fadilah81@gmail.com; 5 Bioinformatics Core Facilities, Institute of Medical Education and Research Indonesia (IMERI), Faculty of Medicine, Universitas Indonesia, Jakarta 10430, Indonesia; 6 Southeast Asian Ministers of Education Organization Regional Centre for Food and Nutrition (SEAMEO RECFON), Jakarta 13120, Indonesia; 7 Department of Community Medicine, Faculty of Medicine, Universitas Indonesia, Jakarta 10430, Indonesia; dhanasari.vt@gmail.com

**Keywords:** gut microbiota, nutrient intake, stunted children

## Abstract

Background: Stunting can be linked to various factors, one of which is dysbiosis. This study aims to analyze the microbiota composition and related contributing factors of stunted and non-stunted children in the slum areas of Jakarta. Methods: The subjects in this study included 21 stunted (HAZ ≤ −2SD) and 21 non-stunted children (−2SD ≤ HAZ ≤ 3SD) aged 2–5 years. Microbiota analysis was performed by extracting DNA from the subjects’ feces and then via 16S rRNA sequencing using next-generation sequencing (NGS). Results: The results of this study showed that in stunted children, the abundance of Mitsuokella (24,469 OTUs), Alloprevotella (23,952 OTUs), and *Providencia alcalifaciens* (861 OTUs) was higher, while in non-stunted children, that of Blautia (29,755 OTUs), Lachnospiraceae (6134 OTUs), Bilophila (12,417 OTUs), Monoglobus (484 OTUs), *Akkermansia muciniphila* (1116 OTUs), *Odoribacter splanchnicus* (42,993 OTUs), and *Bacteroides clarus* (8900 OTUs) was higher. Differences in microbiota composition in the two groups were influenced by nutrient intake, birth history, breastfeeding history, handwashing habits before eating, drinking water sources, and water sources for other activities. Conclusions: This study highlights that stunted children have a significantly different gut microbiota composition compared to non-stunted children, with higher levels of pathogenic bacteria and lower levels of beneficial bacteria. Future research should focus on interventions that can improve the gut microbiota composition to prevent stunting in children.

## 1. Introduction

Jakarta is a national capital with 445 slum settlements [1]. Slum areas are often associated with high rates of malnutrition, which is a problem that still occurs in Jakarta. Based on data, children under five with severe stunting (6.1%) and stunting (1.5%) have been observed in Jakarta [2].

Stunting is a form of malnutrition that is defined as a height that is more than two standard deviations below the World Health Organization (WHO) Child Growth Standards median [3]. It is the result of chronic or recurrent undernutrition, usually associated with poverty, poor maternal health and nutrition, frequent illness, and/or inappropriate feeding and care in early life [4]. Stunting can cause both short- and long-term consequences.

Slum areas are usually inhabited by people with a relatively low socioeconomic status, with drinking water sources contaminated through wastewater disposal allowing the spread of disease [1]. Slum areas are also synonymous with nutritional problems in children, such as stunting [5,6]. However, there are still many children with good nutritional status in these areas (i.e., they are non-stunted).

Recent studies have discussed the relationship between gut microbiota and nutritional status. A longitudinal birth cohort study in Indian children examining persistent stunting and no stunting from 3 to 24 years of age showed that there was an increase in diversity indices with increasing age in all children. The microbiota of control children was enriched in the probiotic species *Bifidobacterium longum* and *Lactobacillus mucosae*, whereas that of stunted children was enriched in inflammagenic taxa [7]. Other studies also showed that disruption of nutritional status in children is not only caused by the abundance of possible pathogenic microbial groups but is also the result of a reduction in several commensal genes [8]. Similar studies in healthy and malnourished children in Bangladesh showed that in malnourished children, the bacterial population of the phylum *Proteobacteria* is higher and that of *Bacteroidetes* is lower in malnourished children than in healthy children [9].

The diversity of the gut microbiota is influenced by many factors such as dietary intake and the environment. Several studies have shown that different areas affect the type of gut microbiota [10,11,12]. Research conducted on healthy Indian people showed profound microbiota differences between rural and urban areas [10]. Similar results were also found in studies of children in urban slums in Bangladesh and the upper-middle-class suburban United States (US), which showed differences in gut microbiota diversity between the two groups [12].

Diet is an important factor influencing the composition of the gut microbiota. Investigations carried out on children in rural areas (Burkina Faso) and urban areas (Italy) with differences in diet showed that this caused differences in the gut microbiota [11]. Similar studies in European and rural African children also reported differences in the composition of the gut microbiota [13].

Some existing data show that even in the same slum environment and with the same economic status, there are groups of children with good nutritional status. It would be interesting to study how parents take advantage of existing environmental limitations and spend limited funds on their children’s nutritional resources, as these behaviors may influence the diversity of the gut microbiota, thereby influencing the child’s tolerance for food and the resistance to various pathogens, which can also indirectly influence their growth. The hypothesis of this study is that there are associations between gut microbiota composition and stunting incidence in the slum areas of Jakarta. The general objective of this study is to provide knowledge on the associations between gut microbiota composition and stunting incidence and related contributing factors among children in the slum areas of Jakarta. By analyzing the factors above regarding the positive impact on the growth of children living in slum areas, the results obtained could be used to inform health practitioners and parents and thus prevent stunting in the future.

## 2. Materials and Methods

### 2.1. Study Protocols and Participants

This cross-sectional study was conducted in one slum urban village in North Jakarta. The subjects of this study were 42 children aged 2–5 years; they were divided into stunted (HAZ ≤ −2SD) and non-stunted (−2SD ≤ HAZ ≤ 3SD) groups. They were not administered antibiotics for a period of 1 month before stool analysis. The study protocol was approved by the Ethical Committee of the Faculty of Medicine, Universitas Indonesia, number KET 22/UN2.F1/ETIK/PPM.00.02/2021.

The questionnaire’s details are reported in the Supplementary Materials; it consisted of questions regarding the sociodemographic information of the subjects, dietary intake, and anthropometric data. Sociodemographic information about the subjects consisted of subject characteristics and factors related to gut microbiota composition (delivery mode, exclusive breastfeeding history, illness history, vaccine history, and source of water). Dietary intake was recorded using the Semi-Quantitative Food Frequency Questionnaire (SQ-FFQ) [14], which can assess usual intake over a long period of time and can estimate portion size.

In this study, microbiota composition analysis was conducted (I). The microbiota compositions of the stunted and non-stunted groups (C) were compared. Based on this, differences in microbiota composition between the two groups (O) were determined.

The sample size needed to assess the bacterial ratio between stunted and non-stunted children was estimated based on this equation [15]:

$$n_{1}=n_{2}=2{\left[\frac{\left(Z_{\alpha }+Z_{\beta }\right)SD}{X_{1}-X_{2}}\right]}^{2}$$

where *n*_1_ = *n*_2_ = the minimal sample size; *Z_α_* = 1.96 for an α of 0.05; *Z_β_* = 0.842 for 80% power; *X*_1_ − *X*_2_ = the mean between groups; and *SD* = the standard deviation between groups.

Based on the reference, the standard deviation of the number of bacterial genera in healthy children was obtained, which was used as a standard deviation for calculation of the sample size. For the mean difference (x_1_ − x_2_), the researchers used an estimate of 1 unit. The results of the minimum sample size calculation for the four genera used are presented in Table 1.

Based on the calculation, to assess the ratio of bacteria, the minimum sample size was 18 samples for each group. With a 10% dropout rate, the total minimum sample for each group was 20 samples. In this study, 42 samples were selected, which were divided into 21 stunted and 21 non-stunted samples.

### 2.2. Fecal Sample Collection, DNA Extraction, and 16S rRNA Sequencing

A DNA/RNA Shield Fecal Collection Tube was given to each subject’s mother. The stool samples were stored at room temperature because the DNA/RNA Shield^TM^ Fecal Collection Tube ensures sample stability during storage at ambient temperature. The DNA/RNA Shield^TM^ reagent effectively lyses samples and inactivates pathogens [17]. DNA extraction was performed using the Presto^TM^ Stool DNA Extraction Kit Protocol from Geneaid (New Taipei City, Taiwan). An A260/A280 ratio of 1.8–2.0 was used to assess DNA purity [18]. The V3-V4 region of the 16S rRNA gene was PCR-amplified based on NGS Metagenomic Amplicon Sequencing. 16S rRNA metagenomic sequencing was carried out using an Illumina Miseq System (San Diego, CA, United States) [19].

### 2.3. Gut Microbiota Composition

Computational analyses were performed using QIIME (http://www.qiime.org, accessed on 8 October 2022) and R Studio. Quality filtering was performed using default settings in QIIME. Reads were grouped into operational taxonomic units (OTUs) based on 97% identity. Representative sequences were then classified by taxonomy using the Silva database as a reference. Taxonomy was added to the OTU table [20]. Singletons and OTUs with an abundance lower than 0.005% were removed [21].

In this study, the Chao1, Simpson, and Shannon indices were used to determine alpha diversity. The Chao1 index was used to describe species richness, which quantifies the actual number of species and takes ultra-low-abundance species into account. The Shannon diversity index (Shannon index) was used to measure both species number (richness) and the distribution of the abundance (evenness). Alpha diversity can also be measured from the Simpson index, which describes how many different types of species are present in a community (group) and the degree to which the population of each species is evenly distributed. As the number of different species increases and the population distribution of species becomes more even, the diversity index increases. A high score indicates high diversity.

Beta diversity is defined as a measure of the similarity or dissimilarity between stunted and non-stunted groups. Beta diversity is determined by principal coordinates analysis (PCoA), which was conducted to determine whether the gut microbiota was grouped or deviated between stunted and non-stunted groups. The Bray–Curtis index was used as the distance method, and for statistical analysis, PERMANOVA was used.

For microbial differences between the stunted and non-stunted groups, linear discriminant analysis (LDA) effect size (LEfSe) was performed. The Kruskal–Wallis rank sum test was applied to detect differential features between assigned taxa, and the LDA was used to quantify the effect size of each feature with a significance alpha value of less than 0.05.

### 2.4. Nutrient Intake

Dietary intake over the past month was assessed using the Semi-Quantitative Food Frequency Questionnaire (SQ-FFQ). Energy intake and macronutrient intake estimated from the SQ-FFQ were comparable to those estimated with 24-h dietary recall. Nutrient intake was calculated using Nutrisurvey 2007. The food composition data were mainly sourced from the Indonesian food composition database and The United States Department of Agriculture (USDA) food composition database. Then, nutrient intake data were compared with the Indonesia Recommended Dietary Allowance (RDA/AKG) for children aged 24–47 and 48–60 months. The RDA for children is shown in Table 2.

### 2.5. Statistical Analysis

Data were analyzed using the Statistical Program Package for Social Science (SPSS) version 20.0. Subject characteristics, parent characteristics, delivery mode, exclusive breastfeeding history, illness history, vaccination history, and WASH are presented in the form of univariate data. Before determining the statistical test used, a normality test was first carried out using the Shapiro–Wilk method because the number of subjects was fewer than 50 children. The association between nutrient intake and gut microbiota composition was analyzed using Spearman’s rank correlation. The associations between other factors and the composition of gut microbiota were analyzed using the MANOVA test. 

## 3. Results

### 3.1. Subject Characteristics

Subject characteristics are shown in Table 3. 

### 3.2. Nutrient Intake

In the present study, the intake of energy and nutrients was determined based on the results of interviews using the Semi-Quantitative Food Frequency Questionnaire (SQ-FFQ), which was confirmed with a 1 × 24-h recall. Based on the data in Table 4, overall energy and macronutrient (carbohydrates, protein, and fat) intake [15] and micronutrient (Zn and Fe) intake in the stunted group were lower than those in the non-stunted group (Table 3). Statistically, there were also significant differences in energy, macronutrient, and zinc intake between the two groups.

### 3.3. Other Factors

The results of other factors can be seen in Table 5. The stunted children tended to become ill more often than the non-stunted children. However, the two groups were comparable regarding the other factors. A total of 81.0% of children in the non-stunted group and 57.1% of children in the stunted group were born vaginally. The number of children who were exclusively breastfed in the stunted group tended to be higher than that in the non-stunted group. Vaccine history included data regarding the completeness of basic vaccine administration. The data in Table 5 show that the number of stunted children who have been completely vaccinated was lower than that in the non-stunted group.

Hygiene and sanitation included personal hygiene, which was assessed by the handwashing habits of the children before eating and their handwashing habits after defecation. In the stunted group, 66.7% of the children did not wash their hands before eating. Likewise, in the non-stunted group, the percentage of children who did not wash their hands before eating was slightly higher than that of those who were used to washing their hands. Another personal hygiene practice is the habit of handwashing after defecating. The habit of handwashing was practiced by the mothers of the children. Based on the results of this study, it was found that most of the subjects’ mothers washed their hands after their children had defecated, with rates of 90.5% for the stunted group and 95.2% for the non-stunted group.

In addition to hygiene data, data on sanitation were also collected. The sanitation aspects observed were sources of drinking water, sources of water for other activities, and places used for defecation. Sources of drinking water consisted of branded gallons of water and refilled water from refill stations. The data in Table 5 show that the predominant source of drinking water, both in the stunted and non-stunted groups, was refilled water, with percentages of 85.7 and 61.9%, respectively. Water is used not only for drinking purposes but also for other activities, such as cooking. The results show that the primary sources of water for other activities in the stunted and non-stunted groups were the Regional Drinking Water Company (PDAM) (90.5%) and well water.

### 3.4. Gut Microbiota Composition

#### 3.4.1. Alpha and Beta Diversity

**Figure 1:**
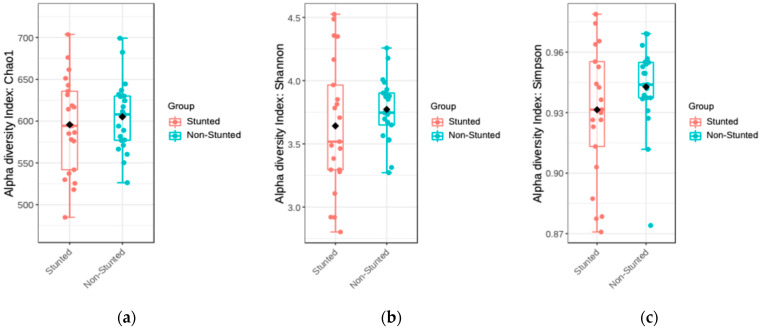
Alpha diversity of gut microbiota of stunted and non-stunted groups. (**a**) Chao1, (**b**) Shannon, and (**c**) Simpson indices.

Alpha diversity is a condition of the diversity of bacteria that exist in one group. This diversity in the stunted and non-stunted groups was analyzed using the Chao1, Simpson, and Shannon indices (Figure 1). 

The Chao1 index describes the richness of bacteria that are in the same group. Figure 1 shows this index in the stunted and non-stunted groups, demonstrating that the richness in the non-stunted group was higher than that in the stunted group. However, based on the results of statistical tests, there was no difference in richness between the stunted and non-stunted groups (*p* = 0.548).

**Figure 2:**
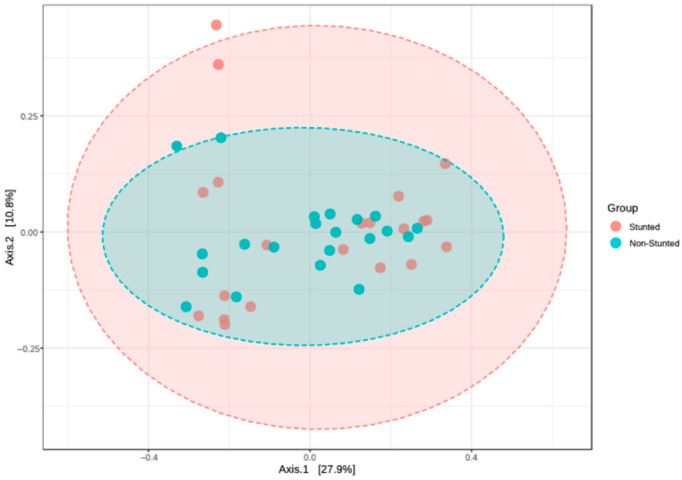
Beta diversity analysis using principal coordinates analysis (PCoA)–Bray–Curtis.

Beta diversity was used to describe gut microbiota variation between the stunted and non-stunted groups. Figure 2 shows that the overall compositions of the microbiota in the stunted and non-stunted groups did not significantly differ (*p* = 0.6). PCoA plots reflect the composition differences between the stunted and non-stunted groups. Axis 1 demonstrates 27.9% dissimilarity between both groups, and Axis 2 shows 10.8% dissimilarity between both groups. The total of Axes 1 and 2 is below 50%. This means that the stunted and non-stunted groups have low dissimilarity. However, in this figure, the non-stunted group is more concentrated than the stunted group, which is spread out.

#### 3.4.2. Gut Microbiota Compositions between the Stunted and Non-Stunted Group

**Figure 3:**
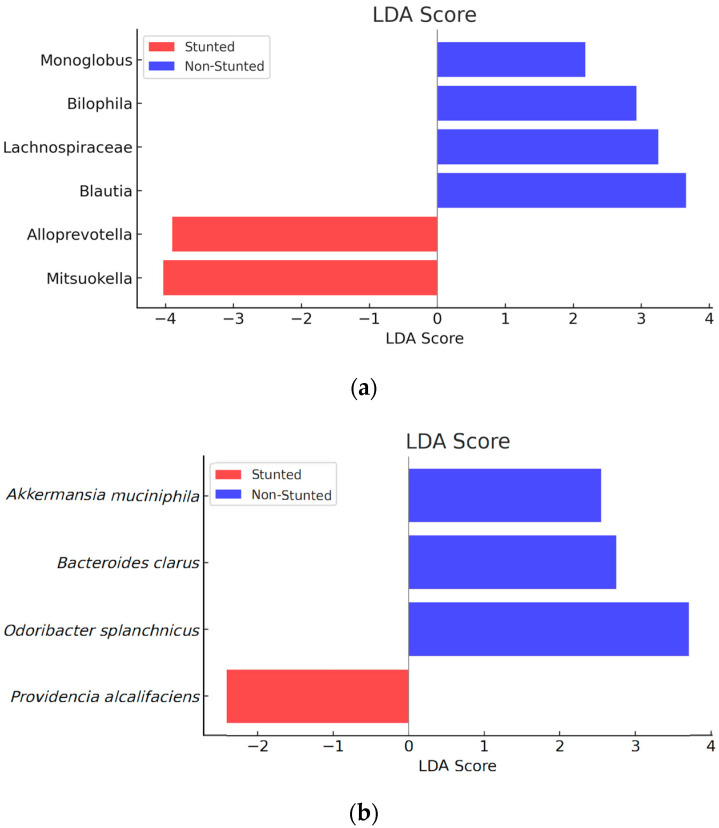
Linear discriminant analysis (LDA) effect size (LEfSe). (**a**) Genera and (**b**) species levels.

According to the results, two genera and one species were significantly different in the stunted group, whereas four genera and two species were significantly different in the non-stunted group. Figure 3 provides an overview of the results of the LEfSe at the genera and species levels, and the abundance of microbiota can be seen in Table 6.

The graph shows that there were significant differences in the compositions of the bacterial genera in the stunted and non-stunted groups. In the stunted group, the most abundant genera were *Mitsuokella* (*p* = 0.037) and *Alloprevotella* (*p* = 0.049). The non-stunted group was dominated by the genera *Blautia* (*p* = 0.016), *Lachnospiraceae* (*p* = 0.048), *Bilophila* (*p* = 0.031), and *Monoglobus* (*p* = 0.030). At the species level, there were also differences in the abundance of bacteria in the stunted and non-stunted groups. In the stunted group, the most abundant bacteria were *Providencia alcalifaciens* (*p* = 0.023). In the non-stunted group, they were *Akkermansia muciniphila* (*p* = 0.012), *Odoribacter splanchnicus* (*p* = 0.040), and *Bacteroides clarus* (*p* = 0.045).

### 3.5. Association between Nutrient Intake and Gut Microbiota Composition

There was an association between energy and nutrient intake and the abundance of several bacteria. Energy intake was related to the abundance of the genera *Mitsuokella*, species of *Odoribacter splanchnicus*, and species of *Providencia alcalifaciens*. Carbohydrate intake was significantly related to the species *Odoribacter splanchnicus*. Protein intake was significantly related to the *Monoglobus* genus, *Odoribacter splanchnicus* species, and *Providencia alcalifaciens* species. Fat intake was significantly associated with the *Lachnospiraceae* genus, *Mitsuokella* genus, *Odoribacter splanchnicus* species, and *Providencia alcalifaciens* species.

**Figure 4:**
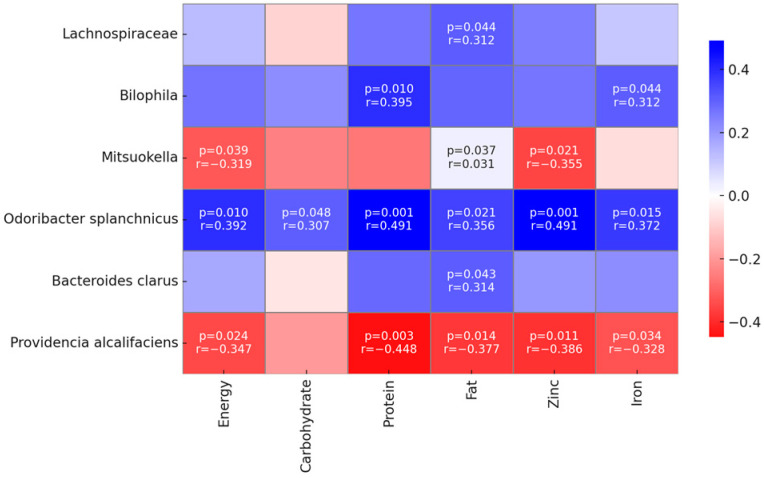
Association between nutrient intake and gut microbiota composition. A blue color indicates a positive association, and a red color indicates a negative association.

Zinc and iron intake also showed an association with the abundance of several bacteria. Zinc was positively correlated with *Odoribacter muciniphila* and negatively correlated with *Mitsuokella* and *Providencia alcalifaciens*. Iron was also positively correlated with *Odoribacter splanchnicus* and *Bilophila* and negatively correlated with *Providencia alcalifaciens*. The overall associations between nutrients and the composition of the microbiota can be seen in Figure 4.

**Figure 5:**
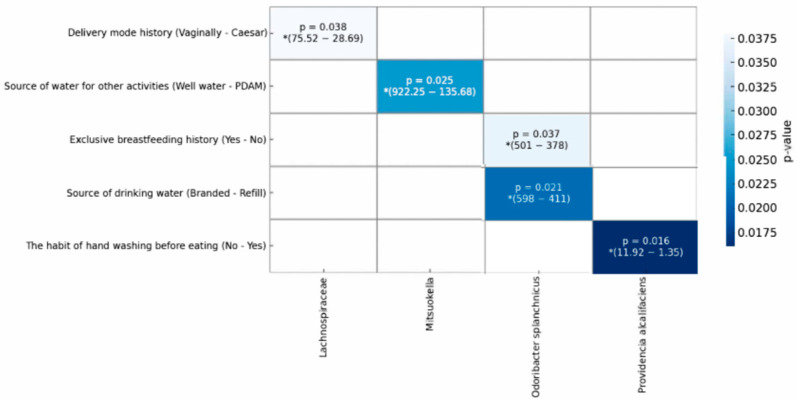
Associations of delivery mode, breastfeeding history, hygiene, and sanitation with gut microbiota composition. * = Average abundance of microbiota in each category (OTU).

The composition of the gut microbiota in the human intestine can vary between individuals, and the diversity of the gut microbiota can be influenced by various factors. In the present study, an analysis was conducted to determine whether there were effects of several variables on the composition of the gut microbiota using multivariate analysis (MANOVA), which showed that illness history, vaccine history, place of defecation, and handwashing habits after defecation had no effect on the composition of the microbiota. However, mode of delivery, exclusive breastfeeding history, handwashing before eating, sources of drinking water, and sources of water for other activities were associated with several types of microbiota (Figure 5).

## 4. Discussion

In the current study, based on LEfSe analyses at the genera and species levels, differences in the types of bacteria between the stunted and the non-stunted groups were found. Apart from several microbiota that were also found in previous research [24], in this study, there was an abundance of *Providencia alcalifaciens* in the stunted group, which was identified as pathogenic bacteria [25,26]. On the other hand, in the non-stunted group, there was an abundance of *Akkermansia muciniphila*, which have the potential to act as probiotics [27]. These findings provide additional information regarding bacteria that have the potential to improve the health of children’s digestive tracts as an effort to enhance nutritional status in children under five.

Previous research regarding the relationship between microbiota and the incidence of stunting in children under five was carried out in two districts in two different provinces in Indonesia. It is known that *Prevotella 9* is positively correlated with children’s linear growth, and the number of these bacteria was lower in the stunted group than in the non-stunted group [24]. Other studies in Asia (China, Japan, Taiwan, Indonesia, and Thailand) showed that in school children, the gut microbiota was dominated by *Prevotella* (P-Type) bacteria [28].

Unlike previous research, this research was conducted on stunted and non-stunted groups in one area, namely, a slum area of Jakarta. In this study, it was found that in the stunted group, there were more pathogenic bacteria such as *Mitsuokella* at the genera level and *Providencia alcalifaciens* at the species level. *Providencia alcalifaciens* is a bacterial pathogen that is associated with diarrhea [25]. Case reports also show that *Providencia alcalifaciens* was found in approximately 7–18 patients in the event of foodborne infections in Fukui, Japan, in 1996 [25]. In addition, *Providencia alcalifaciens* was also found in patients who experienced diarrhea during an outbreak in Kenya in 2013 [26]. In the non-stunted group, more beneficial bacteria were found, such as those of the genus *Lachnospiraceae* and the species *Akkermansia muciniphila*. The health effects of *Akkermansia muciniphila* include strengthening gut integrity, modulating insulin resistance, and protecting the host from metabolic inflammation. Based on this, *A. muciniphila* has potential as an agent for therapy [27]. In line with other research in India regarding the gut microbiota in children with varying nutritional status, stunted children are dominated by inflammatory taxa such as the genus *Desulfovibrio* and the order Campylobacterales, while non-stunted children are dominated by probiotic bacteria such as *Bifidobacterium longum* and *Lactobacillus mucosae* [8].

Dietary intake is one of the factors influencing the composition of the gut microbiota. The type and amount of nutrients consumed can affect the formation of microbiota. Based on a data analysis, energy intake and macronutrients were related to the abundance of several microbiota. A high fat intake can generally reduce carbohydrate intake [29,30]. In the present study, it was also found that the level of fat was higher than that of carbohydrates. Carbohydrate intake had a positive correlation with the *Odoribacter splanchnicus* species. Odoribacter is a group of bacteria that produce short-chain fatty acids (SCFAs) [31], which originate from intestinal microbiota fermentation of non-digestible foods. SCFAs play a role in maintaining health. In the present study, increasing carbohydrate intake was shown to increase the abundance of *Odoribacter splanchnicus* bacteria.

The results also show that protein intake had a positive correlation with the bacteria *Bilophila*, *Odoribacter splanchnicus*, and *Providencia alcalifaciens*. In this study, it was also found that the protein intake of most subjects in the stunted and non-stunted groups was above their nutritional needs. A study showed that high protein intake can increase the risk of inflammatory bowel syndrome (IBS) [29]. *Bilophila* and *Providencia alcalifaciens* are bacteria that have inflammatory properties, which may increase the risk of inflammation when protein intake is increased. A study showed a positive correlation between animal protein intake and *Odoribacter splanchnicus* species. In our study, milk consumption (animal protein) in both subject groups was quite high. This may be a factor in the positive correlation between protein intake and *Odoribacter splanchnicus*.

Fat intake had a positive correlation with the genera *Lachnospiraceae* and *Mitsuokella* and the species *Odoribacter splanchnicus*, *Bacteroides clarus,* and *Providencia alcalifaciens*. Previous studies have shown that a high-fat diet can change the composition of the gut microbiota. Another study showed that in a group of women who consumed a high-fat diet, there was an increase in the abundance of *Lachnospiracea* bacteria. An increased fat intake is also reportedly associated with increased inflammation [29]. This study demonstrated a positive correlation with inflammatory and pathogenic bacteria such as *Mitsuokella* and *Providencia alcalifaciens*.

As well as macronutrients, micronutrient intake (Zn and Fe) was also correlated with several microbiota. In this study, Zn intake was positively correlated with good bacteria (*Odoribacter splanchnicus*) and negatively correlated with pathogenic bacteria (*Mitsuokella* and *Providencia alcifaciens*). Likewise, iron intake had a positive correlation with *Odoribacter muciniphila*, a positive correlation with pathogenic bacteria (*Bilophila*), and a negative correlation with pathogenic bacteria (*Providencia alcalifaciens*).

Zinc and iron are essential minerals for maintaining host health. A low zinc intake is a significant risk factor for poor health, with women and children being the most vulnerable populations. In vivo studies suggest that zinc deficiency alters the gut microbiome composition, with decreased biodiversity, increases in inflammatory markers, and decreases in functional potency being involved in gut–brain signaling. As with Zn, iron deficiency also leads to changes in the composition of the microbiota. The composition of the microbiota, especially probiotics, should be balanced to counteract the side-effects of fortification and thus increase Zn and Fe absorption [32]. A study showed that iron fortification increased *Enterobacteria* and decreased *Lactobacilli* in anemic African children [33,34].

The diversity and abundance of gut microbiota are influenced by several factors, including the method of birth [35,36], exclusive breastfeeding [37,38], illness history, and hygiene and sanitation [39]. In the present study, the results showed that delivery mode, a history of exclusive breastfeeding, washing hands before eating, sources of drinking water, and sources of water for other activities had an impact on several types of microbiota. Delivery mode influenced the composition of the genus *Lachnospiraceae*, with the average abundance in children born vaginally being higher than that in children born by Cesarean section. The results of the present study are in line with the observational results of overweight pregnant women with a normal mode of delivery for their children. These children had more abundant *Lachnospiraceae* than those from normal-weight mothers [40]. However, the present study did not record the mothers’ weights at the time of delivery.

Exclusive breastfeeding is one of the factors that influence the formation of the gut microbiota. In this study, exclusive breastfeeding had an effect on the abundance of *Odoribacter splanchnicus* species, and the average number of children who were exclusively breastfed was higher than that of children who were not. Children who are exclusively breastfed have an abundance of *Bifidobacterium* bacteria. With increasing age, this abundance decreases [38]. The *Odoribacter splanchnicus* species is a group of bacteria that produce short-chain fatty acids, but the amount of these bacteria in human breast milk still needs to be determined.

The hygiene and sanitation factors that influenced the composition of the microbiota in this study were washing hands before eating, sources of drinking water, and sources of water for other activities. Washing hands before eating affected the abundance of *Providencia alcalifaciens* species, which was higher in children who do not wash their hands before eating. It is known that *Providencia alcalifaciens* is a pathogenic bacteria. One of the benefits of good personal hygiene is that it can prevent contamination from pathogenic bacteria.

In the present study, it was found that sources of drinking water influenced the abundance of *Odoribacter splanchnicus* species. Branded gallons of water were used more often more than refilled water. Drinking water can contain both beneficial and pathogenic bacteria. In this case, it is necessary to carry out further research regarding whether branded gallons of water contain *Odoribacter splanchnicus* bacteria. Another influential sanitation factor for the abundance of *Odoribacter splanchnicus* species was the source of drinking water for other activities. The average number of *Odoribacter splanchnicus* species in PDAM water was higher than that in well water.

Overall, in this study, it was found that in children who were delivered vaginally and exclusively breastfed, the abundance of beneficial microbiota was higher than that in those born via Cesarean section and who were non-exclusively breastfed. Likewise, from the perspective of hygiene and sanitation, in children who washed their hands before eating, the abundance of pathogenic microbiota was lower than that in children who did not wash their hands. Drinking water from branded gallons and using PDAM water for other activities resulted in a higher abundance of good bacteria compared to drinking refilled water and using water from wells for other activities. This suggests that when there is a change in habitual behavior, it is possible to improve the composition of the gut microbiota.

The findings of this study may have implications for the prevention and management of stunting. Differences in microbiota, such as a higher abundance of *Akkermansia Muciniphila* in the non-stunted group, indicate a protective effect that can be used in therapeutic interventions [41] In addition, the presence of pathogenic bacteria such as *Providencia alcalifaciens* in stunted children may indicate the role of dysbiosis in stunting [42]. A strategy that can be used to prevent stunting is modulating the digestive tract microbiota, which can be carried out through regulation of nutrient intake, exclusive breastfeeding, and better hygiene and sanitation practices.

A strength of this study is that it was conducted to observe the effects of the composition of the microbiota in stunted and non-stunted children in slum areas. This research was conducted in Kebon Bawang Urban Village, North Jakarta, based on data from the Central Statistics Bureau (BPS), which categorized this village as “light slums” in 2017. In addition, an analysis was also carried out to observe how the associations of several factors (especially energy, macronutrient, and micronutrient intake) with the composition of the microbiota caused differences between the stunted and non-stunted groups. Therefore, the results of this research could encourage the stunted population to adopt behaviors similar to those of the non-stunted population in order to improve their nutritional status.

However, this study also has limitations. In this research, the number of samples used was smaller (21 stunted and 21 non-stunted children) compared to those in previous similar studies (78 stunted and 58 non-stunted children) [24]. However, in this study, significant differences were found in several bacteria, both at the genus and species levels, which could be biomarkers for improving the composition of the gastrointestinal microbiota of stunted children. Apart from that, this research was not carried out longitudinally; therefore, we were unable to observe how changes in the gastrointestinal microbiota under stunting conditions over time could be used to improve the nutritional status of children under five.

## 5. Conclusions

This study found differences between stunted and non-stunted groups with regard to several microbiota. The stunted group was more dominated by pathogenic bacteria such as *Enterococcus*, *Escherichia coli*, *Mitsuokella*, and *Providencia alcalifaciens*. On the other hand, the non-stunted group had higher levels of beneficial bacteria such as *Bifidobacterium*, *Lactobacillus*, and *Akkermansia muciniphila*. These differences were influenced by the mode of delivery, exclusive breastfeeding, handwashing before eating, sources of drinking water, and nutrient intake. Further research is needed to explore the interactions between the microbiota, micronutrient bioavailability, and environmental factors (such as sources of water) to develop effective strategies for the prevention and treatment of stunting.

## Figures and Tables

**Table 1:** Sample size to assess the ratio of bacteria in stunted and non-stunted children.

Genus	Standard Deviation	References	N
*Bifidobacterium*	0.76	Helmyati S. et al., 2017 [16]	9.05
*Lactobacillus*	0.96	Helmyati S. et al., 2017 [16]	14.45
*Enterobacter*	0.73	Helmyati S. et al., 2017 [16]	8.35
*E Coli*	1.07	Helmyati S. et al., 2017 [16]	17.95

**Table 2:** Recommended dietary allowance for children.

Nutrients	24–47 Months *n* = 34	48–60 Months *n* = 8
Energy (Cal) ^a^	1350	1400
Protein (g) ^a^	20	25
Fat (g) ^a^	45	50
Carbohydrate (g) ^a^	215	220
Fe (mg) ^b^	5.8	6.3
Zinc (mg) ^b^	3.4	4.0

Source: ^a^ Indonesian RDA (AKG) based on the Ministry of Health [22]; ^b^ estimated average requirement [23].

**Table 3:** Subject characteristics.

Descriptives	Stunted (n = 21)	Non-Stunted (n = 21)	*p*-Value ^1^
Gender			
Male	9 (42.9%)	14 (33.9%)	0.126
Female	12 (57.1%)	7 (66.7%)	
Age (month) ^1^	37.95 ± 8.71	39.43 ± 10.78	0.628
Weight (kg) ^1^	11.47 ± 1.49	14.36 ± 2.31	<0.001
Height (cm) ^1^	85.90 ± 4.88	95.83 ± 6.45	<0.001
HAZ ^2^	−2.40 (−3.60–(−2.10))	−0.60 (−1.00–2.70)	<0.001

^1^ Analyzed by the independent *t*-test for normally distributed data and by the Mann–Whitney test for non-normally distributed data; ^2^ height for age.

**Table 4:** Nutrient intake of the stunted and non-stunted groups.

Nutrient	Stunted (n = 21)	Non-Stunted (n = 21)	*p*-Value
Energy (Cal) ^b^ [15]	1043 ± 191	1266 ± 178	0.003
Carbohydrate (g) ^a^ [15]	142.7 ± 35.2	165.4 ± 26.9	0.024
Proteins (g) ^a^ [15]	32.2 ± 7.9	39.9 ± 8.9	0.005
Fat (g) ^a^	37.3 ± 8.9	48.4 ± 11.1	0.001
Zn (mg) ^a^	3.5 ± 0.9	4.9 ± 1.4	0.001
Fe (mg) ^b^	6.5 ± 1.9	6.6 ± 1.8	0.734

^a^ Analyzed using the independent *t*-test for normally distributed data and ^b^ the Mann–Whitney test for non-normally distributed data.

**Table 5:** Other variables in the stunted and non-stunted groups.

Variable	Stunted n (%)	Non-Stunted n (%)	*p*-Value ^a^
Delivery Mode			
Vaginal	12 (57.1)	17 (81.0)	0.09
Caesarean section	9 (42.9)	4 (19.0)	
Exclusive Breastfeeding History			
Yes	15 (71.4)	13 (61.9)	0.518
No	6 (28.6)	8 (38.1)	
History of Illness			
No	15 (71.4)	20 (95.2)	0.041
Yes	6 (28.6)	1 (4.8)	
Vaccine History			
Complete	14 (66.7)	17 (81.0)	0.298
Incomplete	7 (33.3)	4 (19.0)	
Source of Drinking Water			
Branded Gallon of Water	3 (14.3)	8 (38.1)	0.083
Refilled Water	18 (85.7)	13 (61.9)	
Source of Water for Other Activities			
PDAM	19 (90.5)	19 (90.5)	1.00
Well water	2 (9.5)	2 (9.5)	
Handwashing Before Eating			
Yes	7 (33.3)	10 (47.6)	
No	14 (66.7)	11 (52.4)	0.351
Handwashing After Defecation			
Yes	19 (90.5)	20 (95.2)	0.554
No	2 (9.5)	1 (33.3)	

^a^ Analyzed by the Chi square test.

**Table 6:** The abundance of gut microbiota in stunted and non-stunted groups.

Microbiota	Abundance (OTU)		*p*-Value ^a^
	Stunted	Non-Stunted	
Blautia	11,550	20,755	0.016
Lachnospiraceae	2601	6134	0.048
Monoglobus	183	484	0.030
Bilophila	10,790	12,417	0.031
Mitsuokella	24,469	2847	0.037
Alloprevotella	23,952	7888	0.049
*Akkermansia muciniphila*	405	1116	0.012
*Odoribacter_splanchinus*	32,747	42,993	0.040
*Bacteroides clarus*	7772	8900	0.045
*Providencia alcalifaciens*	861	353	0.023

^a^ Analyzed by independent *t*-test.

## Data Availability

The data presented in this study are available upon request from the corresponding author.

## Supplementary Materials

The following supporting information can be downloaded at www.mdpi.com/xxx/s1/, the questionnaires.
