# Supplementary material for: Association of Gut Microbiota Composition with Stunting Incidence in Children under Five in Jakarta Slums
Source: Nutrients. 2024 Oct 11;16(20):3444. doi: 10.3390/nu16203444 (PMC11510009; doi:10.3390/nu16203444)
Supplement: Supplementary file 1 [file nutrients-16-03444-s001.zip › nutrients-3232489-supplementary.pdf]

KUESIONER

"The Associations Between Gut Microbiota Composition and Nutritional Status Among Children in Slum Area of Jakarta with Associated Contributing Factors"

Informasi Dasar

**No**	**Keterangan**	
101	Nama Anak:	
102	Jenis Kelamin ☐<br>1. Laki-laki 2. Perempuan	
103	Tanggal Lahir:	
104	Nama Ayah:	
105	Nama Ibu	
106	Alamat:	
107	Nama Interviewer:	
108	Tanggal wawancara:	

Sosio-Demografi Responden

**No**	**Keterangan**	
201	Jumlah anggota keluarga:	
202	Pendidikan Ayah ☐<br>1. Tidak sekolah<br>2. Tamat SD<br>3. Tamat SMP<br>4. Tamat SMU<br>5. Perguruan Tinggi	
203	Pendidikan Ibu ☐<br>1. Tidak sekolah<br>2. Tamat SD<br>3. Tamat SMP<br>4. Tamat SMU<br>5. Perguruan Tinggi	
204	Pekerjaan Ayah ☐<br>1. Tidak bekerja/IRT<br>2. Buruh<br>3. Wiraswasta/pedagang	

	4. Pegawai swasta<br>5. PNS<br>6. Lainnya,……………….	
205	Pekerjaan Ibu ☐<br>1. Tidak bekerja/IRT<br>2. Buruh<br>3. Wiraswasta/pedagang<br>4. Pegawai swasta<br>5. PNS<br>6. Lainnya,……………….	
206	Rata-rata pendapatan per bulan: Rp………………	

Form Pengeluaran

	**Pengeluaran Pangan (IDR)**	
**Kode**	**Jenis Pengeluaran**	**Mingguan**
E.1.	Makanan pokok (beras, jagung, tepung terigu, tepung beras, dll)	
E.2.	Umbi-umbian (singkong, ubi jalar, kentang, sagu, dll)	
E.3.	Ikan (ikan segar, ikan asin, udang, cumi, kepiting, kerrang, dll)	
E.4.	Daging (daging sapi, ayam, bebek, hati, dendeng, jeroan, daging kaleng dll)	
E.5.	Telur dan susu (telur aya, te;ur bebek, telur puyuh, susu segar, susu bubuk, susu anak, keju, dll)	
E.6.	Sayuran (bayam, ketimun, wortel, kol, toge, buncir, kacang Panjang, dll)	
E.7.	Kaacang-kacangan (kacang tanah, kacang kedele, kacang hijau, tahu, tempe, tauco, dll)	
E.8.	Buah-buahan (jeruk, pisang, papaya, semangka, rambutan, manga, dll)	
E.9.	Minyak dan lemak (minyak kelapa, minyak sakit, margarin, mentega, santan, kelapa dll)	
E.10.	Bahan minuman (gula pasir, gula merah, the kopi, coklat sirup , dll)	
E.11.	Bumbu (garam, jahe, kunyit, penyedap/vetson, kecap, asam, ketumbar dll)	
E.12.	Konsumsi lainnya (mie instan, bihun, kerupuk, agar dll)	
E.13.	Makanan dan minuman jadi (roti, biscuit, kue, bakso, gorengan, sate, lontong, soto, makanan ringan, jus, minuman bersoda, air kemasan, air gallon, es krim dll	

E14.	Tembakau, sirih (rokok, tembakau, sirih, dll)	
**E.15.**	**Sub total (E1 s.d. E14)**	
E	**Rerata pengeluaran pangan sebulan (E15x (30/7))**	

	**Pengeluaran Non Pangan**			
**Kode**	**Jenis pengeluaran**	**Mingguan (a)**	**Bulanan (b)**	**Tahunan ©**
F.1.	Perumahan (sewa rumah, kontrak, dll)			
F.2.	Pemeliharaan/perbaikan rumah			
F.3.	Rekening listrik, gar, air/PDAM, minyak tanah dll			
F.4.	Pulsa, internet dll			
F.5.	Alat mandi (sabun, pasta gigi, shampoo dll)			
F.6.	Kosmetik (beak, lipstick, dll)			
F.7.	Biaya pendidikan			
F.8.	Biaya kesehatan (dokter/bidang, RS, puskesmas, obat-obatan dll)			
F.9.	Pakaian dll			
F.10.	Barang tahan lama (AC, HP, TV, motor dll			
F.11.	Pajak, asuransi			
F.12.	Biaya pesta (Pernikahan, khitan, ulang tahun)			
F.13.	Biaya rekreasi			
F.14.	Biaya transportasi			
F.16.	Rerata Sebulan	F15a x (30/7)	F15b =	F15 c/12
F	Rerata Pengeluaran Non Pangan Sebulan = F15a+F15b+F15c			

Total Pengeluaran Sebulan = E + F = Rp ……………….

Total Pengeluaran Sebulan/anggota keluarga = (E + F)/jml anggota keluarga = Rp ………………

Faktor yang mempengaruhi komposisi mikrobiota usus

No	Keterangan	
301	Cara Kelahiran ☐<br>1. Normal 2. Cesar	
302	Apakah anak mendapatkan ASI eksklusif ☐<br>1. Ya 2. Tidak	
303	Sampai usia berapa anak mendapatkan ASI?	
304	Usia (bulan) anak pertama kali mendapatkan makanan tambahan:	
305	Apakah anak minum susu formula? ☐<br>1. Ya 2. Tidak	
306	Pada usia berapa anak pertama kali minum susu formula?	
307	Apakah saat ini anak masih minum susu formula? ☐<br>1. Ya 2. Tidak	

Riwayat Sakit
Apakah anak menderita penyakit dibawah ini selama 2 minggu terakhir

No	Nama Penyakit	Ya/Tidak	Lama sakit (hari)	Pengobatan*
401	Diare			
402	Tifus			
403	Batuk			

***pengobatan:**

1. sembuh sendiri
2. minum oralit
3. mengobati sendiri
4. datang ke puskesmas
5. datang ke RS
6. Lainnya…

Sumber air

No	Sumber air	Ket
501	Darimana sumber air untuk minum di rumah anda?<br>1. PDAM<br>2. Air gallon (bermerk)<br>3. Air gallon isi ulang<br>4. Air hujan<br>5. Air sumur<br>6. Lainnya:	
502	Darimana sumber air untuk keperluan selain minum?<br>1. PDAM<br>2. Air hujan<br>3. Air sumur<br>4. Air sungai<br>5. lainnya	
503	Apakah anda/anak anda minum air yang tidak dimasak<br>Alasan:	

Higiene dan Sanitasi

No	Keterangan	
601	Apakah anda/anak anda menggunakan tangan atau sendok saat makan<br>1. Tangan 2. Sendok ☐	
602	Apakah anak anda mencuci tangan sebelum makan<br>1. Ya 2. Tidak ☐	
603	Apakah anda mencuci tangan sebelum memberi makan<br>1. Ya 2. Tidak ☐	
604	Dimana anak anda biasanya BAK/BAB?<br>1. Toilet pribadi<br>2. Toilet umum<br>3. Sungai	
605	Apakah anak anda mencuci tangan ketika selesai dari kamar mandi?<br>1. Ya 2. Tidak ☐	
606	Apakah anda mencuci tangan ketika selesai dari kamar mandi?<br>1. Ya 2. Tidak ☐	

607	Dimana biasanya anda membuang sampah rumahtangga?<br>1. Petugas sampah ☐<br>2. Sungai<br>3. Disembarang tempat<br>4. Dibakar	

Riwayat vaksinasi

No	Keterangan	Jawaban
701	Apakah anak anda mendapatkan vaksin dasar lengkap?<br>Alasan:	
702	Dimana anak anda biasanya divaksin	

Riwayat ke Posyandu dan Faskes

No	Keterangan	
801	Apakah anda datang ke posyandu untuk penimbangan?<br>Alasan:	
802	Apakah anda datang ke puskesamas/rumah sakit untuk berobat?<br>Alasan:	

Pengukuran Antropometri

No	Keterangan	
901	Tinggi Badan (cm):	
902	Berat Badan (kg)	

Nama Subyek : …………………………. Nama enumerator: …………..
Umur : …………………………. Hari/tgl wawancara: ………….
Nama ibu subyek : ………………………….
Alamat : ………………………….

**No**	**Nama Bahan Makanan**	**Frekuensi**			**Porsi**	
		**Hari**	**Minggu**	**Bulan**	**URT**	**Gram**
**I**	**Makanan Pokok**					
	Nasi					
	Kentang					
	Roti					
	Singkong					
	Jagung					
	Ubi jalar					
	Mie					
	Tepung					
	Lainnya:					
**II**	**Protein Hewani**					
	Daging sapi					
	Daging kambing					
	Daging ayam					
	Ikan segar					
	Ikan asin					
	Produk laut (udang, cumi, kerang)					
	Hati sapi					
	Hati ayam, ampela, usus					
	Telur					
	Lainnya					
**III**	**Protein Nabati**					
	Tempe					
	Tahu					
	Oncom					
	Kacang tanah					
	Kacang hijau					
	Kacang merah					
	Lainnya:					
**No**	**Nama Bahan Makanan**	**Frekuensi**			**Porsi**	

		Hari	Minggu	Bulan	URT	Gram
**IV**	**Sayuran**					
	Bayam					
	Kangkung					
	Daun singkong					
	Wortel					
	Kol					
	Tauge					
	Sawi hijau					
	Lainnya:					
**V**	**Buah-buahan**					
	Pisang					
	Papaya					
	Jeruk					
	Semangka					
	Melon					
	Nanas					
	Mangga					
	Apel					
	Duku					
	Anggur					
	Klengkeng					
	Salah					
	Pir					
	Jambu					
	Buah naga					
	Lainnya:					
**VI**	Susu dan olahannya					
	Susu					
	Keju					
	Es krim					
	Yoghurt					
	Lainnya					
**VII**	**Minyak**					
	Minyak kelapa					
	Minyak goreng					
	Mentega					
**No**	**Nama Bahan Makanan**	**Frekuensi**			**Porsi**	

		Hari	Minggu	Bulan	URT	Gram
	Margarin					
	Lainnya					
**VIII**	**Serba-serbi snack**					
	Agar-agar					
	Coklat					
	Biscuit					
	Siomay					
	Otak-otak					
	Bakso					
	Lainnya:					
**IX**	**Minuman**					
	Teh manis					
	Sirop					
	Jus buah					
	Minuman kemasan					
	Lainnya:					

Nama Responden :
Umur :
Enumerator :
Hari/Tgl.Wawancara :

**Jenis Makan**	**Waktu Makan**	**Nama Makanan/Minuman**	**Metode Persiapan**	**Bahan Makanan**	**Merk Makanan/Minuman (jika ada)**	**Berat Porsi**	
**Sarapan**						**URT****	**Gram**
**Snack**							
**Makan siang**							
**Snack**							
**Makan malam**							

***lingkari salah satu; **URT = ukuran rumah tangga**
